# Prolactin gene expression in primary central nervous system tumors

**DOI:** 10.1186/1477-5751-12-4

**Published:** 2013-01-14

**Authors:** Graziella Alebrant Mendes, Júlia Fernanda Semmelmann Pereira-Lima, Maria Beatriz Kohek, Geraldine Trott, Marlise Di Domenico, Nelson Pires Ferreira, Miriam da Costa Oliveira

**Affiliations:** 1 Postgraduate Program in Pathology, Universidade Federal de Ciências da Saúde de Porto Alegre (UFCSPA), Porto Alegre, Brazil; 2 Center of Neuroendocrinology, Complexo Hospitalar Santa Casa, Porto Alegre/RS, CEP, 90020-090, Brazil; 3 Biomedicine Student, UFCSPA, Porto Alegre, Brazil

**Keywords:** Prolactin, CNS tumors, immunohistochemistry, quantitative real-time PCR, gene expression

## Abstract

**Background:**

Prolactin (PRL) is a hormone synthesized in both the pituitary gland and extrapituitary sites. It has been associated with the occurrence of neoplasms and, more recently, with central nervous system (CNS) neoplasms. The aim of this study was to evaluate prolactin expression in primary central nervous system tumors through quantitative real-time PCR and immunohistochemistry (IH).

**Results:**

Patient mean age was 49.1 years (SD 15.43), and females accounted for 70% of the sample. The most frequent subtype of histological tumor was meningioma (61.5%), followed by glioblastoma (22.9%). Twenty cases (28.6%) showed prolactin expression by immunohistochemistry, most of them females (18 cases, 90%). Quantitative real-time PCR did not show any prolactin expression.

**Conclusions:**

Despite the presence of prolactin expression by IH, the lack of its expression by quantitative real-time PCR indicates that its presence in primary tumors in CNS is not a reflex of local production.

## Background

PRL was initially associated with mammary development and lactation. However, more than 300 biological functions have been attributed to this hormone [1]. The human PRL gene is located on chromosome 6 and its expression is not limited to the pituitary gland, occurring also in such extrapituitary sites as endometrium, decidua, myometrium, T lymphocytes, leukocytes, brain, prostate, skin and adipose tissue [2,3]. The gene transcription is further regulated by two independent promoters: a proximal pituitary PRL promoter and a distal extrapituitary PRL promoter [4].

Recent studies have suggested an association of PRL with tumor proliferation, evidencing the hormone as an antiapoptotic or mitogenic factor [1]. Studies showed that PRL extends the lobular-alveolar cells survival on the lactating mammary gland, inhibiting apoptosis and stimulating the proliferation of many cell lines of breast cancer [5]. *In vivo* studies showed that high levels of PRL accelerated the emergence of spontaneous mammary tumors [6] and could act as an anti-cytotoxic factor in breast cancer cells, contributing to drug resistance [7]. PRL is also involved in prostate tumor growth, acting as a survival factor for epithelial cells, and PRL expression was correlated with tumor differentiation degree [8-10]. In addition, hyperprolactinemia was associated with a worse prognosis in patients with colon and rectum cancer [11].

Regarding the CNS, there are studies showing the presence of hyperprolactinemia, intracellular PRL and PRL receptor (PRL-R) in different types of CNS tumors [12,13], and intracellular PRL and serum PRL were associated with a tendency of increased vascular density and significant increase of cell proliferation markers Ki-67 and Mcm-2, suggesting that PRL may play a role in the development of these tumors [14].

The purpose of this study was to assess PRL expression in primary CNS tumors by real-time PCR and to correlate these findings with immunohistochemical hormone detection, contributing to a better understanding of the interrelationship between PRL and CNS tumors.

## Results

The study included 70 patients: 49 (70%) females and 21 (30%) males. Their mean age was 49.1 years (SD 15.43). The mean age of the group with positive IH (54.90 years) was statistically significantly different (p = 0.046) from that of the group with negative IH (46.78 years).

As for pathological diagnosis, the most frequent histological subtype was meningioma (43 cases, 61.5%), followed by glioblastoma (16 cases, 22.9%), astrocytoma (5 cases, 7.2%) oligoastrocytoma and anaplastic oligodendroglioma (2 cases each, 2.8%), anaplastic ependymoma (1 case, 1.4%), and glioma (1 case, 1.4%). Regarding tumor location, 43 cases (61.5%) were located in the meninges, 26 cases (37.1%) were supratentorial, and 1 case (1.4%) infratentorial.

**Figure 1:**
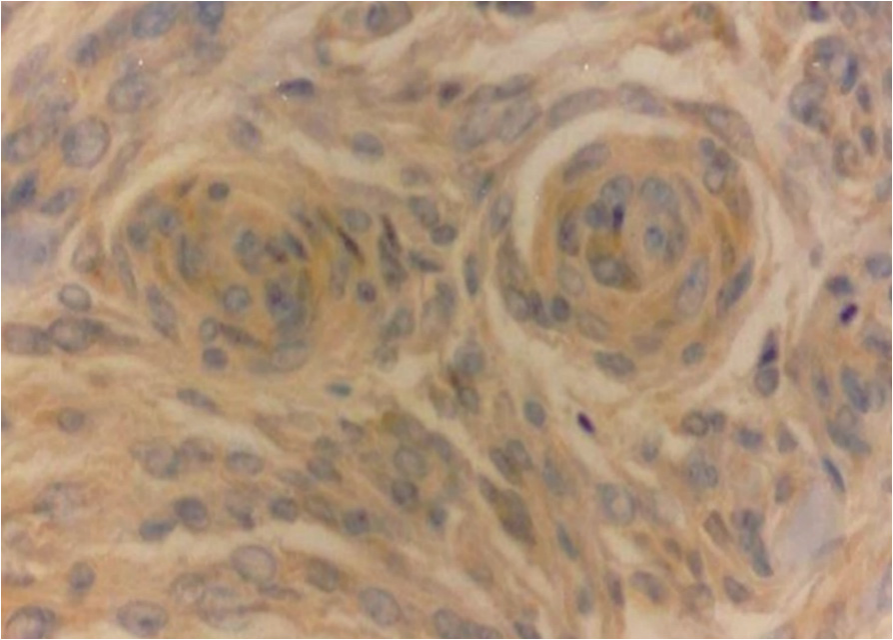
Meningioma showing PRL immunopositivity in meningothelial cells (400x).

**Figure 2:**
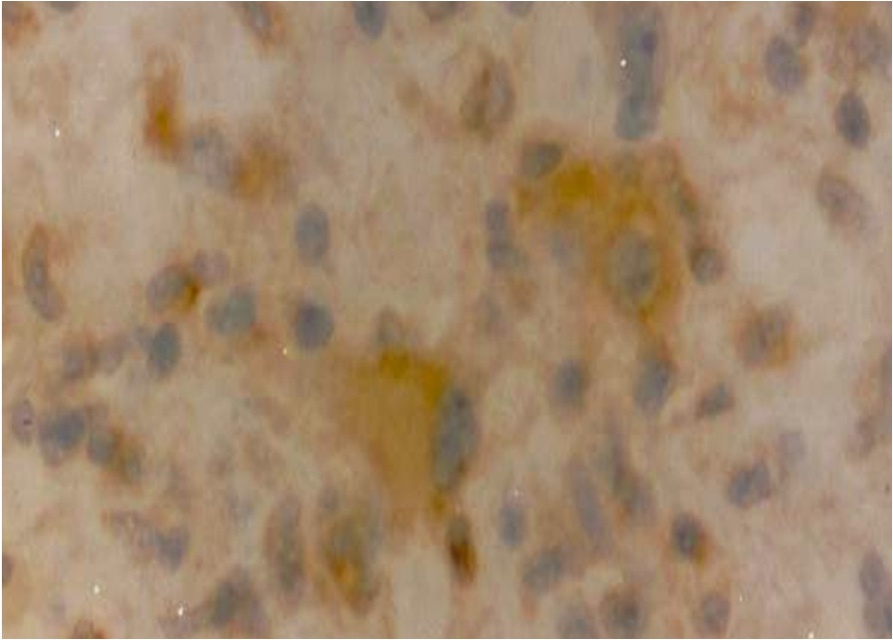
Glioblastoma with PRL immunopositivity (400x).

Regarding IH expression of intracellular PRL, 20 cases were positive, representing 28.6% of the sample with a predominance of females (18 cases, 90%). Of the positive cases, 15 were meningiomas (transitional meningioma, 8 cases, 40%; meningeal meningioma, 5 cases, 25%; psammomatous meningioma, 1 case, 5%; secretory meningioma, 1 case, 5%), one was oligoastrocytoma (5%) and 4 were glioblastomas (20%) (Table 1). See Figure 1 and 2. Statistical significance was obtained by relating gender (18 females versus 2 males) to IH PRL positivity (p = 0.043). There was no statistically significant relationship of IH PRL positivity with age (< 40 and ≥ 40 years), pathological diagnosis, and tumor location (Table 2).

**Table 1:** Sex, age, histological diagnosis, location and grade of primary CNS tumors in patients with positive immunohistochemistry for PRL

**Case**	**Sex**	**Age (years)**	**Histological diagnosis**	**Location**	**Grade**
**1**	F	74	Transitional meningioma	Meninge	I
**2**	F	51	Glioblastoma	Supratentorial	IV
**3**	F	64	Transitional meningioma	Meninge	I
**4**	F	53	Meningeal meningioma	Meninge	I
**5**	F	69	Meningeal meningioma	Meninge	I
**6**	F	49	Psammomatous meningioma	Meninge	I
**7**	F	60	Transitional meningioma	Meninge	I
**8**	F	84	Glioblastoma	Supratentorial	IV
**9**	F	57	Transitional meningioma	Meninge	I
**10**	F	55	Meningeal meningioma	Meninge	I
**11**	F	52	Meningeal meningioma	Meninge	I
**12**	F	37	Glioblastoma	Supratentorial	IV
**13**	F	47	Secretory meningioma	Meninge	I
**14**	F	34	Transitional meningioma	Meninge	I
**15**	F	59	Meningeal meningioma	Meninge	I
**16**	F	63	Glioblastoma	Supratentorial	IV
**17**	F	37	Transitional meningioma	Meninge	I
**18**	M	62	Transitional meningioma	Meninge	II
**19**	M	34	Oligoastrocytoma	Supratentorial	II
**20**	F	57	Transitional meningioma	Meninge	I

Sex: F=females; M=males; PRL=prolactin.

**Table 2:** Rates of positivity for PRL by immunohistochemistry

		**PRL IH (+)**		
**Variable**	**n**	**nº**	**%**	**p**
Age, years				0.386[1]
<40	21	4	19.0	
≥40	49	16	32.7	
Sex				0.043[1]
males	21	2	9.5	
females	49	18	36.7	
Histological type				0.461[2]
Meningioma	43	15	34.9	
Glioblastoma	16	4	25.0	
Astrocytoma	5	0	0.0	
Others	6	1	16.7	
Location				0.356[2]
Meninges	43	15	34.9	
Supratentorial	26	5	19.2	
Infratentorial	1	0	0.0	

PRL: prolactin; IH: immunohistochemistry; [1]: Chi-Square Test (Yates); [2]: Fisher’s exact test; nº: number of cases.

Quantitative real-time PCR failed to show any PRL expression in both normal CNS tissue and CNS tumor samples.

## Discussion

The current series is characterized by primary CNS tumor-bearing patients whose ages were similar to those of patients in other studies [15,16]. Meningiomas prevailed in association with female predominance [17]. Regarding location, most meningiomas in our sample occurred at different locations as compared to most tumors described in the literature [18].

In our study, 28.6% of the sample presented positive IH to PRL. Unlike Abech and colleagues’ findings [14], PRL was positive in older patients in our series. In 1999, positive IH for PRL was described in a patient with gangliocytoma [19].

Hyperprolactinemia and presence of intracellular PRL and PRL-R have been detected by immunohistochemistry in different types of CNS tumors. Cicarelli and colleagues [12] found hyperprolactinemia and presence of PRL-R in 27.2% and 45.4% of meningiomas and 61.5% and 69.2% of schwannomas, without correlation of hyperprolactinemia with PRL-R. Leães and colleagues [13] identified hyperprolactinemia in 30.5%, presence of intracellular PRL in 21.9% and PRL-R in 39% of 82 cases, with positive correlation of serum PRL with presence of intracellular PRL. A recent study by Abech and colleagues [14] revealed presence of intracellular PRL in 45.6% of neuroepithelial tumors and meninges and elevated serum PRL in 33.9% of the cases. Therefore, our findings of positive intracellular PRL are similar to the those of the literature. The meaning of intracellular presence of PRL may vary according to the malignant potentials of the tumors, and there is no data in the current literature on the possible causes for the presence of positive PRL immunohistochemistry in different types of CNS tumors. However, in gliomas of different grades, Scott and colleagues [20] found a significant increase in the expression of Ki-67, Mcm-2 and cyclin A and B1 as tumor grade increased, and it is speculated that PRL, like the above-mentioned proteins, may be related to more aggressive tumors [1]. Ducret and colleagues [21] demonstrated increased intracellular calcium in glioblastoma cells induced by the presence of PRL, increased thymidine incorporation, cell growth and half-life. Regarding meningiomas, Jimenez-Hakim and colleagues [22] demonstrated that PRL concentrations stimulated the growth of meningiomas significantly.

A single report utilizing conventional PCR detected PRL mRNA expression in the human hypothalamus, pituitary and cerebellum [23]. In studies with rats, PRL mRNA was detected in various degrees in the hypothalamus, cerebellum, caudate, brain stem, amygdala, thalamus, hippocampus and cortex [24,25]. In this study, no expression of PRL was identified in the pool of 9 samples of CNS peritumoral tissue (gray and white matter and meningothelial tissue) through quantitative real-time PCR. Real-time PCR is the most powerful tool for quantitative nucleic acids analysis. It is widely considered as the gold standard for nucleic acid quantification and has become the method of choice for the detection of mRNA because of its unparalleled amplification and precision capability [26-29]. Sample of prolactinoma was used as positive control, demonstrating that real-time PCR is able to identify PRL expression. It removes the possibility that the technique was not sensitive enough to detect PRL and to corroborate immunohistochemical findings. In general, polyclonal antibodies have high affinity and wide reactivity, becoming more sensible as compared with monoclonal antibodies because they are more likely to identify multiple epitopes of the target protein. However, their specificity is lower as compared to monoclonal antibodies, being more susceptible to cross-reactivity with other antigens [30]. PCR methods generally have a higher sensitivity and specificity than immunohistochemical ones and agreement between results of immunohistochemistry and PCR methods can be low [31-33]. Real time -PCR is the most sensitive method for the detection and quantification of mRNA, especially for low abundance specimens, like RNAs, cells and tissues [27,34]. Real time-PCR assays are 10.000 to 100.000-fold more sensitive than RNase protection assays, 1000-fold more sensitive than dot blot hybridization, detecting a single copy of a specific transcript and is a method with lower variation, usually between 0 and 5% for TaqMan probes [27-29,34,35]. The real time -PCR technique, specially with TaqMan probes, is the best option when analyzing gene with low expression level, from limited samples once high sensitivity and accuracy are needed [27,36]. There is no data in the current literature about PRL gene expression by quantitative real-time PCR in normal samples of the CNS. To the best of our knowledge, there are no studies analyzing PRL gene expression in different types of CNS tumors either by conventional PCR or by quantitative real-time PCR. Therefore, the absence of PRL expression by quantitative real-time PCR in human samples of different types of CNS tumors, the main finding of this study, is an unprecedented one.

So far, the identification of positive PRL by immunohistochemistry suggested possible extrapituitary hormone production, but immunological methods do not allow to discriminate between locally produced PRL and PRL that is captured by dependent or independent mechanism of the PRL-R. The PRL-R is ubiquitously expressed and its isoforms vary across tissues. The long isoform is highly expressed in the choroid plexus, mammary gland, pancreas, adrenal, kidney and intestine. In the brain, with the exception of the choroid plexus, there is a low expression of PRL-R. PRL-R triggers intracellular signaling pathways, the best known of which being the Jak-Stat. In breast cancer cells, PRL activates Jak2, stimulates phosphorylation of Stat1, Stat3 and Stat5 and induces cell proliferation [37,38]. In breast cancer cells and Nb2 cells, PRL stimulates ERK1/2 phosphorylation in the mitogen-activated protein (MAP) kinase pathway, which appears to mediate the effects of PRL on cell proliferation [39,40]. The PRL-R facilitates the action of members of the Src kinase family, activating kinase B protein and the phosphatidylinositol 3-kinase (PI3 kinase) pathway [41]. The interconnection between the different signaling pathways of PRL can increase the proliferation, survival, cell migration and invasion of breast cancer [42] and is responsible for mediating antiapoptotic and proliferative effects of PRL [43]. The absence of PRL detection by quantitative real-time PCR does not favor the hypothesis of local production because, unlike IH, it is a method that analyses the mRNA of protein in order to identify local production of PRL. In a study by Abech and colleagues [14], 45.6% of cases presented positive IH to PRL and 30.6% of cases presented hyperprolactinemia. In addition, in a study carried out by Leães and colleagues [13], of the total number of patients with positive IH to intracellular PRL, 27.8% presented hyperprolactinemia, 38.9% were positive for PRL-R, and 44.4% had neither of the two variables. Although the available data do not allow us to define the origin of positive IH PRL in primary tumors of the CNS, they strongly suggest that it does not reflect local production. It is postulated that the presence of PRL in CNS is due to the transport of pituitary PRL from the blood by the choroid plexus, with subsequent distribution via cerebrospinal fluid and taking up by neurons and glial cells to exert modulatory functions. It is presumed that the PRL receptor or binding protein in the choroid plexus functions as a transporter, enabling circulating PRL to gain access to various brain regions [2,44,45].

## Conclusions

Despite the presence of prolactin expression by immunohistochemistry, the lack of its expression by quantitative real-time PCR indicates that its presence in primary tumors in central nervous system is not a reflex of local production. Thus, further studies are needed regarding the origin of this PRL identified with the immunohistochemistry technique and its true role in the pathogenesis of CNS tumors.

## Materials and methods

Design: Cross-sectional observational study.

### Subjects

Patients with primary diagnosis of CNS tumor who underwent neurosurgery at Hospital São José, Complexo Hospitalar Santa Casa of Porto Alegre, Brazil, from February 2007 to July 2010 were included in this study. Informed consent was obtained from all subjects before participation. Medical records were reviewed to collect data concerning sex, age, tumor location, and anatomopathological diagnosis. The anatomopathological findings were reviewed by a second pathologist.

The study was conducted in a statistically estimated sample of 70 patients. The research project was approved by the Ethics Committee of the institution (263/09).

### Immunohistochemistry

Samples of tumoral tissue were fixed with 10% buffered formalin and embedded in paraffin. Serial 4 μm sections were stained with hematoxylin and eosin and subjected to IH. Polyclonal antihuman antibody (Dako, Carpinteria, CA, code A0569-1, dilution 1:1000) and Advance™ HRT (Dako, Carpinteria, CA, code K4068, ready-to-use) were used for detection of intracellular PRL. Endogenous peroxidase activity was blocked using two baths of 10 minutes in 5% hydrogen peroxide (H_2_O_2_) 30 V in methanol. Unspecific proteins were blocked using 1% BSA for 30 minutes. Incubation with primary antibody was performed overnight at 4°C. Incubation with secondary antibody and tertiary antibody was performed for 40 minutes at room temperature. The same tissues were used as negative controls, incubated with the same antibodies except the primary one, which was replaced by BSA. The antigen-antibody binding was visualized with the DAB chromogen (diaminobenzidin). Counterstaining was with Harris hematoxylin; the slides were dehydrated and mounted in synthetic resin. Human pituitary was used as positive control. Intracellular PRL positivity was based on presence of at least 1% of cells with clearly marked cytoplasm [13,46].

### Quantitative real-time PCR

CNS tumor fragments were obtained immediately after surgery, snap-frozen in liquid nitrogen and kept in biofreezer at −80°C until RNA extraction. The procedure for RNA extraction was performed using TriReagent (Ludwig Biotec, Porto Alegre, RS), according to the manufacturer’s instructions. RNA was reversely transcribed in a final volume of 21μL using Superscript 1ST Strand System for RT-PCR (Invitrogen, San Diego, CA), according to the manufacturer’s instructions. All cDNA samples were quantified using the Nanodrop 1000, measuring absorbance at 260 nm and 280 nm. Complementary DNAs were of good quality when the 260/280 ratio was greater than 1.7. The same procedures were adopted for the *pool* of normal peritumoral tissue as well as the prolactinoma used as positive control.

In order to perform quantitative real-time PCR, all cDNA samples were diluted to a final concentration of 400 ng/μL and amplified using TaqMan gene expression Assay (PRL Hs01062137_m1; GAPDH Hs99999905_m1) and TaqMan Gene Expression Master Mix (4369510) (Applied Biosystems, Foster City, CA) in a total reaction volume of 15μL, under the following conditions: initial denaturation at 50°C for 2 minutes and at 95°C for 10 minutes, followed by 40 cycles at 95°C for 15 seconds and 60°C for 1 minute. The equipment used was the StepOnePlus (Applied Biosystems, Foster City, CA). For relative quantification the 2^-ΔΔCT^ method described by Livak and Schmittgen [47] was adopted. The GAPDH gene was used as reference.A *pool* consisting of 9 samples of normal peritumoral tissue (gray and white matter CNS and meningothelial tissue) was used as calibrator to allow comparison between CNS tumoral and normal samples. A sample of prolactinoma was used as positive control and mixed reagents without the presence of cDNA were used as negative control. The tests with CNS tumor samples were performed in duplicate, and the GAPDH gene and PRL gene were always tested in the same assay, avoiding possible differences between assays. The GAPDH gene was detected in all samples. The PRL gene was detected in the prolactinoma sample (positive control) in all assays. In each reaction plate and for each gene tested, the *pool* of normal CNS tissue samples (calibrator) was performed in triplicate, the positive control (prolactinoma) and negative control in duplicate.

### Statistical analyses

Age-related aspects were first described by mean and standard deviation and comparison between the IH groups (positive and negative) using Student’s t test. Next, age was dichotomized into two groups: over and under 40 years old. Statistical analysis was performed with histological meningioma subtypes, astrocytoma and glioblastoma, grouped in other histological oligoastrocytoma subtypes, anaplastic oligodendroglioma, anaplastic ependymomas and unclassified glioma. Among the positive and negative IH groups, categorical data was described by counts and percentages and comparisons made using the Chi-square test or Fisher’s exact test in the presence of expected values below 5. The level of significance was 5% and the data were analyzed by SPSS 17.0.

## Abbreviations

CNS: Central nervous system; IH: Immunohistochemistry; PRL: Prolactin; PRL-R: PRL receptor.

## Competing interests

The authors declared that they have no competing interest.

## Authors’ contributions

MGA Conception, Planning, Technical execution, Interpretation of results, Drafting of the article. PLJFS Conception, Planning, Technical assistance, Interpretation of results, Review and approval of the final version. KMB Planning, Technical assistance, Interpretation of results, Review of article.TG Responsible for samples, Technical execution. DDM Responsible for samples, Technical execution. HNP Neurosurgeon, Obtaining of samples. OMC Conception, Planning, Interpretation of results, Review of article. All authors read and approved the final manuscript.
